# Potential determinants of low circulating glucagon‐like peptide 2 concentrations in Zambian children with non‐responsive stunting

**DOI:** 10.1113/EP090492

**Published:** 2023-02-06

**Authors:** Ellen Besa, Mizinga Jacqueline Tembo, Chola Mulenga, Monica Mweetwa, Naheed Choudhry, Kanta Chandwe, Chad Storer, Richard Head, Beatrice Amadi, Talin Haritunians, Dermot McGovern, Geoffrey Kwenda, Madusha Peiris, Paul Kelly

**Affiliations:** ^1^ Tropical Gastroenterology and Nutrition Group, School of Medicine University of Zambia Lusaka Zambia; ^2^ Blizard Institute, Centre for Neuroscience, Surgery and Trauma, Barts and The London School of Medicine and Dentistry Queen Mary University of London London UK; ^3^ Genome Technology Access Center at McDonnell Genome Institute Washington University in St Louis St Louis MO USA; ^4^ Cedars‐Sinai Medical Center Inflammatory Bowel and Immunobiology Research Institute Los Angeles CA USA; ^5^ Department of Biomedical Sciences, School of Health Sciences University of Zambia Lusaka Zambia

**Keywords:** enteroendocrine cells, glucagon‐like peptide, malnutrition

## Abstract

Nutrient sensing determines digestive and hormonal responses following nutrient ingestion. We have previously reported decreased levels of glucagon‐like peptide 2 (GLP‐2) in children with stunting. Here we demonstrate the presence of enteroendocrine cells in stunted children and explore potential pathways that may be involved in reduced circulating levels of GLP‐2. At the time of performing diagnostic endoscopies for non‐responsive stunted children, intestinal biopsies were collected for immunofluorescence staining of enteroendocrine cells and transcriptomic analysis. Circulating levels of GLP‐2 were also measured and correlated with transcriptomic data. An exploratory genome‐wide association study (GWAS) was conducted on DNA samples (*n* = 158) to assess genetic contribution to GLP‐2 variability. Intestinal tissue sections collected from non‐responsive stunted children stained positive for chromogranin A (88/89), alongside G‐protein‐coupled receptors G‐protein receptor 119 (75/87), free fatty acid receptor 3 (76/89) and taste 1 receptor 1 (39/45). Transcriptomic analysis found three pathways correlated with circulating GLP‐2: sugar metabolism, epithelial transport, and barrier function, which likely reflect downstream events following receptor–ligand interaction. GWAS analysis revealed potential genetic contributions to GLP‐2 half‐life and receptor binding. Enteroendocrine cell loss was not identified in stunted Zambian children as has been observed for goblet and Paneth cells. Transcriptomic analysis suggests that GLP‐2 has pleiotrophic actions on the intestinal mucosa in malnutrition, but further work is needed to dissect pathways leading to perturbations in nutrient sensing.

## Abstract (highlights)

- **What is the central question of this study?**
  Non‐responsive stunting is characterised by a progressive decline of circulating glucagon‐like peptide 2: what are the possible causes of this decline?
- **What is the main finding and its importance?**
  In contrast with the established loss of Paneth and goblet cells in environmental enteropathy, there was no evidence of a parallel loss of enteroendocrine cells as seen by positive tissue staining for chromogranin A. Transcriptomic and genomic analyses showed evidence of genetic transcripts that could account for some of the variability seen in circulating glucagon‐like peptide 2 values.

## 1 INTRODUCTION

Nutrient sensing is the fundamental process of detecting the availability of substrates necessary for cell growth, proliferation, repair and gut function (Sanchez‐Garrido & Shenoy, 2020). Once detected, nutrient absorption results in the activation of metabolic and sensory signalling pathways in the mucosal layer that produce whole‐body biological responses before nutrient absorption into the circulation (Duca et al., 2021). Intestinal stem cells differentiate into either absorptive enterocytes or secretory cells, which include goblet cells, Paneth cells and enteroendocrine cells (Beumer et al., 2020). Physiological responses to ingested nutrients are orchestrated by more than 12 major enteroendocrine cell (EEC) types expressed throughout the epithelium of the gastrointestinal tract from the stomach to the rectum (Gribble & Reimann, 2016). These cells are single and scattered, comprising about 1% of the overall epithelial cell population, with a lifespan not exceeding 1 week (Worthington et al., 2018). EECs can be open or closed cell types (Latorre et al., 2016); open cells have microvilli extending onto the luminal surface for nutrient sampling while closed cells are located close to the basal membrane, do not reach the lumen of the gut and lack microvilli. Both cell types accumulate their secretory products, mainly peptides/hormones, in cytoplasmic granules, which have either large dense core vesicles (LDCVs) or smaller synaptic‐like microvesicles (SLMVs) similar to those found in neurons (Engelstoft et al., 2015). Components of these vesicles such as chromogranin A, a matrix soluble glycoprotein commonly found in LDCVs, or synaptophysin, a membrane glycoprotein of SLMVs, can be used as general markers of identification. Hormonal release from secretory vesicles is mobilised by elevated concentrations of cytoplasmic calcium and enhanced by cyclic adenosine monophosphate (Worthington et al., 2018).

Enteroendocrine cells can sense specific macronutrients by nutrient flux through metabolic pathways, or by nutrient‐sensing G‐protein‐coupled receptors (GPCRs) (Gribble & Reimann, 2019; Raka et al., 2019). Nutrient sensing GPCRs expressed on EECs transduce external stimuli by generating cascades of intracellular signalling pathways leading to cell activation and subsequent release of peptides/hormones (Reimann et al., 2012; Symonds et al., 2015). The GPCR signalling cascade is such that once the primary stimulus has been sensed via the membrane receptor, the signal has to be conveyed into the cell via a transduction triad (receptor, transducer, effector) involving G‐proteins (De Francesco et al., 2017). Nutrient sensing receptors can be broadly classified into taste receptors, amino acid receptors and free fatty acid receptors (Latorre et al., 2016). Classic taste receptors which are either in the taste receptor type 1 (T1R) or type 2 (T2R) family are expressed in the intestine and colon (Steensels & Depoortere, 2018; Symonds et al., 2015). GPCR119 (G‐protein receptor (GPR) 119) is a GPCR expressed on L‐cells and enterochromaffin cells (Peiris et al., 2018) which has been found to bind specifically to free fatty acid metabolites, particularly phospholipids and fatty acid amide ligands (Furness et al., 2013). Fatty acids are categorised by carbon chain length and are sensed by specific fatty acid receptors which are categorised according to their ligand profile. Short‐chain fatty acids (SCFA) are ligands of free fatty acid receptor 3 (FFAR3) and free fatty acid 2 (FFAR2) (Yonezawa et al., 2013). FFAR3 is a SCFA receptor expressed in intestinal L‐cells and activated by propionate, butyrate and valerate (Ichimura et al., 2014).

Glucagon‐like peptide‐2 (GLP‐2), a 33 amino acid peptide produced and stored in L‐type EECs, has been shown to enhance intestinal growth, digestion, absorption, barrier function and blood flow (Brubaker, 2018). Several studies on patients with ulcerative colitis and Crohn's disease have shown that treatment with teduglutide, a degradation resistant analogue of GLP‐2, resulted in reduced needs for parenteral nutrition in both paediatric and adult patients (Carter et al., 2017; Seidner et al., 2018). Endogenous GLP‐2 is rapidly inactivated by protease dipeptidyl peptidase IV (DPP IV) (Janssen et al., 2013). A study looking at how serum fasting GLP‐1 and GLP‐2 changed with intestinal adaptation in children with paediatric onset intestinal failure (IF) found that GLP‐2 levels were higher in children with IF compared to healthy controls, particularly in those with small bowel–colon continuity (Mutanen & Pakarinen, 2017). We have also shown that in children with diarrhoea, GLP‐2 levels are elevated in acute but not persistent diarrhoea. In malnutrition and enteropathy, GLP‐2 levels progressively declined as the children's growth became more stunted (Amadi et al., 2017; Besa et al., 2020). We set out to test the hypothesis that reduced circulating GLP‐2 levels in children with environmental enteropathy and stunting is attributable to small intestinal EEC loss or reduced function, assessed by immunostaining, transcriptomic and genomic analysis.

## 2 METHODS

### 2.1 Ethics approval

Ethical approval for the study was obtained from the University of Zambia Biomedical Research Ethics Committee (UNZABREC Ref no. 006‐02‐16, dated 3 January 2018) and written informed consent was obtained from the child's parent/guardian. The study conformed to the standards set by the latest revision of the *Declaration of Helsinki* except for registration in a database.

#### 2.1.1 Study participants

**FIGURE 1:**
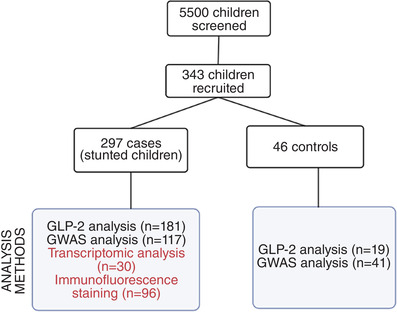
Flow chart showing the number of cases and controls recruited in each arm of the study and the different analysis methods used. Circulating GLP‐2 was assessed on plasma samples, genome wide association studies (GWAS) on DNA samples, and transcriptomic/immunofluorescence analysis on tissue biopsy samples obtained from the cases designated non‐responders. Transcriptomic analysis was performed on 59 biopsies from 30 children.

A study of biomarkers of environmental enteropathy in children (BEECH) was carried out in Misisi compound, Lusaka (Amadi et al., 2021). Enteropathy has been proposed to underlie stunted growth (Harper et al., 2018) and as EE is asymptomatic, we recruited children based on stunted status. Two hundred and ninety‐seven stunted children with weight‐for‐length (WLZ) score < −2 and 46 non‐stunted controls (WLZ > −2) aged between 0 and 18 months were recruited in this study and different analyses were conducted on specific subsets of these children (Figure 1).

Ninety‐six of the children with stunting were declared non‐responsive to nutritional rehabilitation (defined as lack of consistent improvement in length for age *z*‐score (LAZ) to a value > −2, with no clear alternative explanation for growth failure) and were invited to the hospital for further investigations, which included endoscopic biopsies (from the D2 region of the duodenum) for diagnostic and research purposes. Endoscopy was not performed on non‐stunted controls as we had no ethical justification for taking biopsies from healthy children (Chandwe et al., 2021). Weight measurements were done using a mother–child scale (Seca 874, Seca, Hamburg, Germany), length using infantometers (Seca 416) and height using UNICEF height boards. Wasting, stunting and underweight were assessed using WLZ, LAZ and weight for age *z*‐score (WAZ) calculated using the WHO Anthropometry software v.3.2.2.

#### 2.1.2 Blood and tissue collection

Blood samples were collected at recruitment, into BD vacutainer plastic blood collection tubes with EDTA (Becton Dickinson, Plymouth, UK) from both stunted and non‐stunted children (assumed to be in the fed state). A further blood sample was collected from the children designated as non‐responders at the beginning of the endoscopic procedure (therefore in the fasted state). The blood samples were then spun down at 856 *g* for 15 min and the plasma obtained aliquoted and stored at −80°C until analysis. Where possible, tissue samples (*n* = 3 biopsies/patient) were also collected from the duodenum during endoscopy and were either snap‐frozen or oriented using a dissecting microscope and fixed in 10% formal saline for 24 h. The formalin‐fixed tissue was then processed into paraffin blocks and sectioned for immunofluorescence studies.

#### 2.1.3 Methods of analysis

**FIGURE 2:**
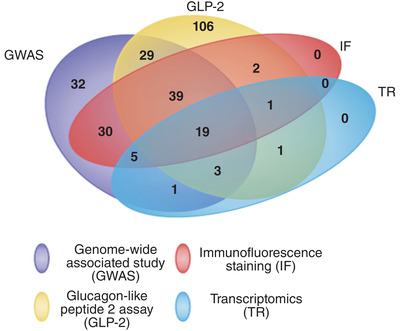
Samples were analysed using transcriptomics (TR), glucagon‐like peptide 2 assays (GLP‐2), immunofluorescence (IF) and a genome wide association study (GWAS). Only 19 children were analysed for all 4, and transcriptomics and immunofluorescence was only done on cases.

Samples collected were analysed using four different methods: enzyme‐linked immunosorbent assays (ELISA; GLP‐2), genome‐wide association study (GWAS), immunofluorescence and bulk RNA sequencing (transcriptome) (Kelly et al., 2021). Tissue samples used for transcriptomic and immunofluorescence analysis were only collected from stunted children who had been designated non‐responders as we could not justify doing endoscopies on children who were responding to nutrition rehabilitation or non‐stunted controls. Nineteen of the samples collected were analysed using all four methods, 48 children had at least three methods of analysis, 63 had two methods used and 138 had a single method of analysis (Figure 2).

#### 2.1.4 Hormone assays

Plasma samples were tested for GLP‐2 by ELISA (EZGLP2‐37K; Millipore Corp., St Charles, MO, USA). GLP‐2 has an active form (1–33) which is cleaved to its inactive form GLP‐2 (3–33) by the protease DPP IV. The sandwich ELISA assay has a specificity of 100% for human GLP‐2 (1–33) and 112% for human GLP‐2 (3–33), and therefore cannot distinguish between the active (GLP‐2 (1–33)) and inactive (GLP‐2 (3–33)) forms. It has a sensitivity of 1 ng/ml and an accuracy of between 75% and 97%.

In view of concerns about specificity, we conducted preliminary experiments to determine cross‐reactivity with other pro‐glucagon gene products. The peptides used in the specificity assay test were GLP‐1 (1–36) (Bachem, Bubendorf, Switzerland, cat. no. H‐6025.0500), oxyntomodulin (Bachem, cat. no. H‐6058.0500, glicentin‐related pancreatic polypeptide (GRPP; Bachem, cat. no. H‐6062.0500) and the GLP‐2 isoforms 1–33 and 3–33 (ChemScene, Monmouth Junction, NJ, USA, cat. no. CS‐7691 and cat. no. CS‐0136913). Specificity was tested using recovery experiments where known amounts of each peptide (0.5, 5, 50, 500 ng/ml) were added to separate aliquots of the assay buffer and measured.

#### 2.1.5 Immunofluorescence

Paraffin embedded sections (3 μm) were incubated at 60°C in a Stuart Scientific (Appleton Woods, UK) S150 orbital incubator for 1 h before being deparaffinised in xylene and ethanol. Antigen retrieval was then carried out using a citrate‐based antigen unmasking solution (Vector Laboratories, Burlingame, CA, USA) diluted 10‐fold and incubated at 92–95°C for 20 min. Permeabilisation was then performed in either phosphate‐ or Tris‐buffered solution (PBS or TBS) containing 0.25% Triton X‐100 (Sigma‐Aldrich, St Louis, MO, USA) at room temperature, after which they were washed in PBS/TBS 4 times for 5 min each. Blocking buffer (Trident Universal protein blocker (GeneTex, CA, USA)/10% goat serum (R&D Syste) was applied for 30 min at room temperature. The tissue was then incubated with primary antibody diluted in 10% goat serum and then washed in PBS/TBS 4 times for 5 min each. The primary antibodies used were as follows: rabbit anti‐chromogranin A (1:50; ab45179, Abcam, Cambridge, UK) (Dlugosz et al., 2014), rabbit anti‐FFAR3/GPR41 (1:50; ab103718, Abcam) (Abdelli et al., 2019), rabbit anti‐GPCR GPR119 (1:100; ab75312, Abcam) (Little et al., 2014) and rabbit anti‐taste receptor type 1 member 1 (TAS1R1; 1:100; ab155143, Abcam) (Zhou et al., 2016). Secondary species‐specific antibody was then added at a dilution of 1:50: goat anti rabbit Alexa fluor 488 (ab150077, Abcam; Cui et al., 2020) was used for chromogranin A and TAS1R1 staining and goat anti rabbit Alexa fluor Cy3 (ab97075, Abcam) (Pu et al., 2015) for GPR119 and FFAR3. The tissue was then incubated for 1 h, before washing in PBS 4 times for 5 min each. Slides were mounted in 4′,6‐diamidino‐2‐phenylindole‐ containing mounting medium (Vectashield mounting medium for fluorescence, Vector Laboratories). A negative control was included in every run. Single staining was done on all the tissue.

#### 2.1.6 Cell counting

Immunofluorescence staining for the different antibodies was done on all the available tissue but only clearly stained images for specific tissue subsets were analysed. The slides were viewed at a magnification of ×20 on an EVOS fluorescence microscope (Thermo Fisher Scientific, Waltham, MA, USA) and images captured using the EVOS Cell Imaging system. For each field of view, grid squares of 10 μm × 10 μm were used to count the number of positive cells. This was then divided by the denominator, that is, the number of squares containing any part of the crypt to obtain an index:

$$\mathrm{Chromogranin}\;A\;\mathrm{positive}\;\mathrm{index}=\frac{\mathrm{Number}\;\mathrm{of}\;\mathrm{chromogranin}\;A\;\mathrm{positive}\;\mathrm{cells}}{\mathrm{Number}\;\mathrm{of}\;\mathrm{grid}\;\mathrm{squares}\;\mathrm{showing}\;\mathrm{crypts}}$$

#### 2.1.7 Transcriptomic analysis

Two small intestinal biopsies collected during endoscopy were immediately snap‐frozen in liquid nitrogen before storage at −80°C. RNA was extracted using Trizol reagents (Thermo Fisher Scientific) followed by silica column purification (RNeasy Mini Kit, Qiagen, Hilden, Germany) and quantified using a Nanodrop spectrophotometer (ND‐2000c, Thermo Fisher Scientific), prior to transporting to the Beijing Genomics Institute (BGI) for library construction and sequencing as previously described (Chama et al., 2019). Gene expression levels were quantified by Expectation Maximization (RSEM) software and expressed in fragments per kilobase of transcript per million (FPKM).

#### 2.1.8 Genotyping and quality control

Saliva from one hundred and sixty‐four subjects was collected using the Oragene DNA kit (DNA Genotek Inc., Kanata, ON, Canada) and sent to Cedars‐Sinai Medical Center (Los Angeles, CA, USA). DNA was extracted using the NucleoSpin Tissue kit (Macherey‐Nagel, Düren, Germany) and genotyped using the H3Africa Consortium Array (Illumina, San Diego, CA, USA; Mulder et al., 2018) following the manufacturers’ protocols. Four samples failed genotyping, and genotyping concordance for sample replicates was >99.98%. Related samples and samples with sex discrepancies were excluded (*n* = 2). One hundred and fifty‐eight DNA samples (117 cases and 41 controls) passed quality control and were available for downstream analyses. Single nucleotide polymorphisms (SNPs) with >3% missing data, minor allele frequency (MAF) <3%, or failing Hardy–Weinberg equilibrium (*P* < 1 × 10^−6^) were excluded.

#### 2.1.9 Data analysis

GLP‐2 concentrations in plasma were determined to be non‐normally distributed using the Shapiro–Wilk test, so data are presented as median and interquartile range (IQR). A *P*‐value <0.05 was considered significant. Data were also analysed using GraphPad Prism 5 (GraphPad Software, San Diego, CA, USA) and STATA v.15 (StataCorp LLC, College station, TX, USA). Missing data were treated as absent, and no imputation was made. Transcriptomic analysis was done by correlating FKPM values for each mRNA transcript with circulating GLP‐2 concentrations. Positively correlated transcripts were then analysed using CompBio software v.2.0 (Gene Technology Access Center and Washington University in St Louis, MO, USA) to enable identification of enriched biological themes. This was supported by Gene Ontology enrichment analysis using g:Profiler software (version e103_eg50_p15_eadf141) (Uku et al., 2019) and pathway enrichment analysis using the pathfindR R package (v.4.0.5) (Ulgen et al., 2019). The g:Profiler tool GOSt was used to map the positively correlated genes to known functional databases and detect statistically over‐represented terms. All options were left default and the results were presented in form of a Manhattan plot. PathfindR utilises active subnetworks to create protein–protein interaction networks in the gene list, which are then used as a primary input to determine what pathway this network is associated with in Biocarta (http://www.biocarta.com/genes/index.asp). The results of this were visualised using the ggplot R package. Cytoscape v.3.9.0 (Shannon et al., 2003) was used to visualise the co‐expressed genes that had a positive correlation coefficient to GLP‐2 greater than 0.5. Genetic association analyses were performed for 99 subjects with data on GLP‐2 levels, using linear regression including principal components for population sub‐structure and case–control status as covariates (PLINKv1.9) (Chisenga et al., 2018). Between 10 and 100 million permutations were further performed for variants associated at *P* < 1 × 10^−4^ (PLINKv1.9). GWAS analysis revealed genetic variants that correlated with baseline circulating GLP‐2 levels (*P* ≤ 0.01). Ensemble and g:SNPense software from Gene Ontology was used to identify correlated SNPs, their consequences and pathways that could potentially be affected by them.

## 3 RESULTS

A total of 200 samples (181 cases and 19 controls) were studied (Table 1). There were more males among the stunted children (59%) than the non‐stunted controls (32%), with the stunted children older than non‐stunted controls. The difference in age was a consequence of the recruitment strategy, which identified controls before 6 months of age.

**TABLE 1:** Baseline characteristics for children recruited as stunted (cases) and non‐stunted (controls).

Participant characteristics	Cases (*n* = 181)	Controls (*n* = 19)
Sex: male, *n* (%)	107 (59)	6 (32)
Age, median (IQR) (months)	11 (7, 14)	4 (3, 5)
Anthropometry: WAZ	−2.57 (−3.14, −2.21)	0.08 (−0.86, 0.55)
Anthropometry: WLZ	−1.23 (−1.86, −0.59)	0.73 (0.21, 1.49)
Anthropometry: LAZ	−2.99 (−3.56, −2.57)	−0.81 (−1.01, 0.17)

*Note*: *z*‐scores are indicated as number of standard deviations (SD) from normal. Abbreviations: LAZ, length for age *z*‐score; WAZ, weight for age *z*‐score; WLZ, weight for length *z*‐score.

### 3.1 Circulating GLP‐2 levels decreased in children progressing to non‐responsive stunting

**FIGURE 3:**
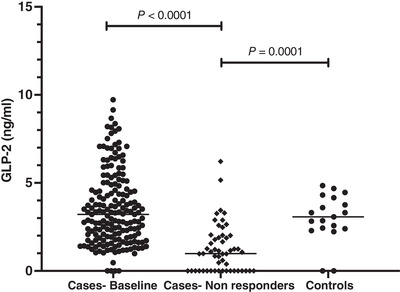
Circulating levels of glucagon‐like peptide 2 in cases (*n* = 181) and controls (*n* = 19) at baseline and in cases designated non‐responders at the time of endoscopy (*n* = 53).

Circulating GLP‐2 concentrations at the time of recruitment in stunted children (3.2 ng/ml, IQR 1.9, 4.9) were comparable to controls (3.1 ng/ml, IQR 2.39, 4.16), but as stunting progressed to the point when non‐response was declared, GLP‐2 levels declined significantly (1.1 ng/ml, IQR 0, 2.03; *P* < 0.0001, Wilcoxon signed rank test) (Figure 3). In the children designated non‐responders, 47% were male with a median age of 18 months (IQR 15, 21) and length‐for‐age *z*‐score of −3.28 SD (IQR −3.90, −2.76).

### 3.2 GLP‐2 ELSA specificity

**FIGURE 4:**
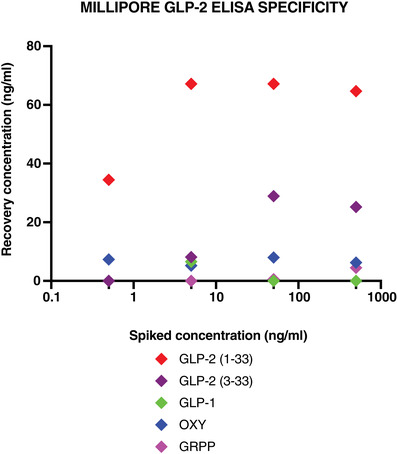
Spike recovery concentrations of pro‐glucagon derived peptides glucagon‐like peptide 2 (GLP‐2) (1–33) and GLP‐2 (3–33), glucagon‐like peptide 1 (GLP‐1), oxyntomodulin (OXY) and glicentin related pancreatic polypeptide (GRPP).

The Millipore Total ELISA kit predominantly detected both isoforms of GLP‐2, with the highest recovery seen in the complete isoform (GLP‐2 (1–33)). There was negligible cross‐reactivity with all other pro‐glucagon derived peptides (Figure 4).

### 3.3 EECs were present in tissue samples from children designated non‐responders

**FIGURE 5:**
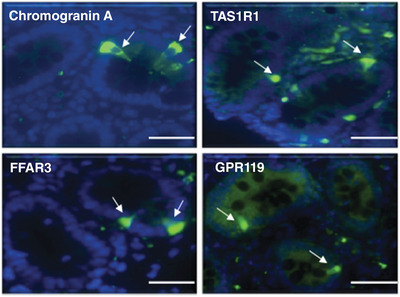
Representative images of intestinal tissue from stunted children designated non‐responders immunostained with chromogranin A, taste 1 receptor 1 (TAS1R1), free fatty acid receptor 3 (FFAR3) and G‐protein receptor 119 (GPR119). The arrows indicate positively stained cells (green); scale bar: 50 μm.

Positive receptor staining was seen for all four antibodies, with chromogranin A staining demonstrating the strongest signal (Figure 5).

The staining of GPR119, FFAR3 and TAS1R1 was indicative of the presence of a range of enteroendocrine cells (Table 2) in stunted children designated non‐responders.

**TABLE 2:** Immunofluorescence staining for enteroendocrine cell presence was only done on tissue samples obtained from cases designated as non‐responders (*n* = 96).

Enteroendocrine cell markers	Proportion of positively stained tissue, *n* (%)	Marker index, median (IQR)
Chromogranin A (*n* = 89)	88/89 (98.9)	0.21 (0.17–0.29)
G‐protein receptor 119 (*n* = 87)	75/87 (86.2)	0.07 (0.03–0.1)
Free fatty acid receptor 3 (*n* = 89)	76/89 (85.4)	0.07 (0.04–0.13)
Taste 1 receptor 1 (*n* = 45)	39/45 (86.6)	0.06 (0.02–0.11)

Marker indices were derived from cell counts (see Section 2). No child was negative for all four stains.

### 3.4 Transcriptomic profiling

**FIGURE 6:**
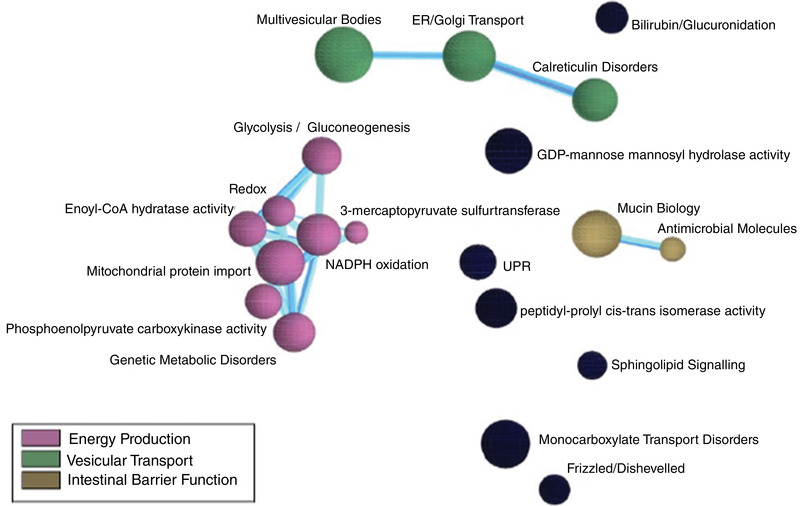
CompBio analysis output showing pathways that positively correlated with circulating plasma GLP‐2 concentrations aggregated into the three major themes.

DNA samples from non‐responsive stunted children showed 1104 gene transcripts were positively correlated with GLP‐2 at a significance level of *P* < 0.05. The CompBio analysis tool identified dominant biological themes in the set of positively correlated gene transcripts (Figure 6). The themes identified ranged from various signalling pathways to sugar transporters, epithelial barrier related, sugar metabolism and antimicrobial peptides, suggestive of the role GLP‐2 plays in three overarching themes, namely metabolism, vesicular transport and intestinal barrier function.

**FIGURE 7:**
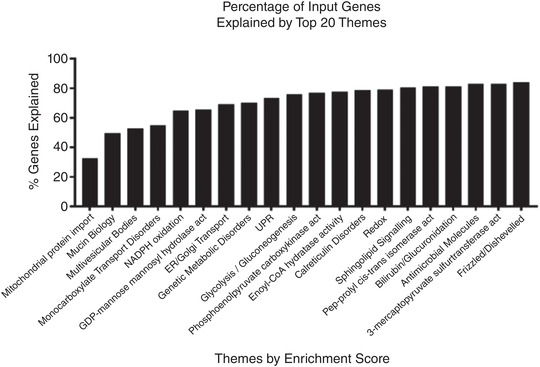
Percentage of genes biologically explained across the top 20 themes in the CompBio analysis, displayed as a cumulative plot.

Transcripts from eight genes (*ATOH1*, *CDX1*, *CDX2*, *ELF3*, *FZD5*, *HES1*, *HNFIB* and *MSI1*) that play a role in the differentiation and maturation of secretory cells were found to be positively correlated with GLP‐2 with correlation coefficients >0.26. The top 20 pathways included over 80% of the genes correlated with circulating GLP‐2 concentrations (Figure 7).

**FIGURE 8:**
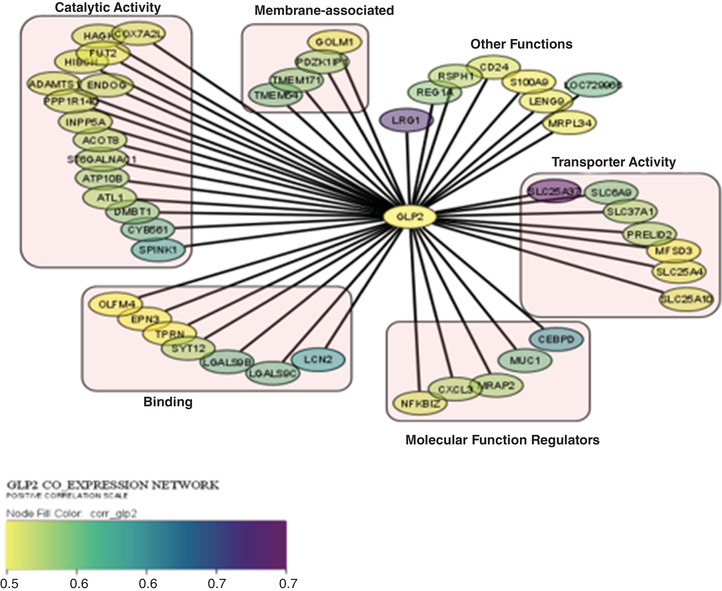
Network of genes correlated with GLP‐2 in non‐responsive stunted children, using Cytoscape. The nodes are mapped continuously according to correlation with GLP‐2 (yellow has the lowest correlation value and purple has the highest).

Cytoscape visualisation grouped the co‐expressed genes according to their protein class with the bulk being involved in catalytic activity and coloured according to their correlation coefficient. The highest correlations were between GLP‐2 and the solute carrier *SLC25A37*, and *LRG1* (Figure 8).

### 3.5 Genetic variants associated with glucagon‐like peptide‐2 concentration

**FIGURE 9:**
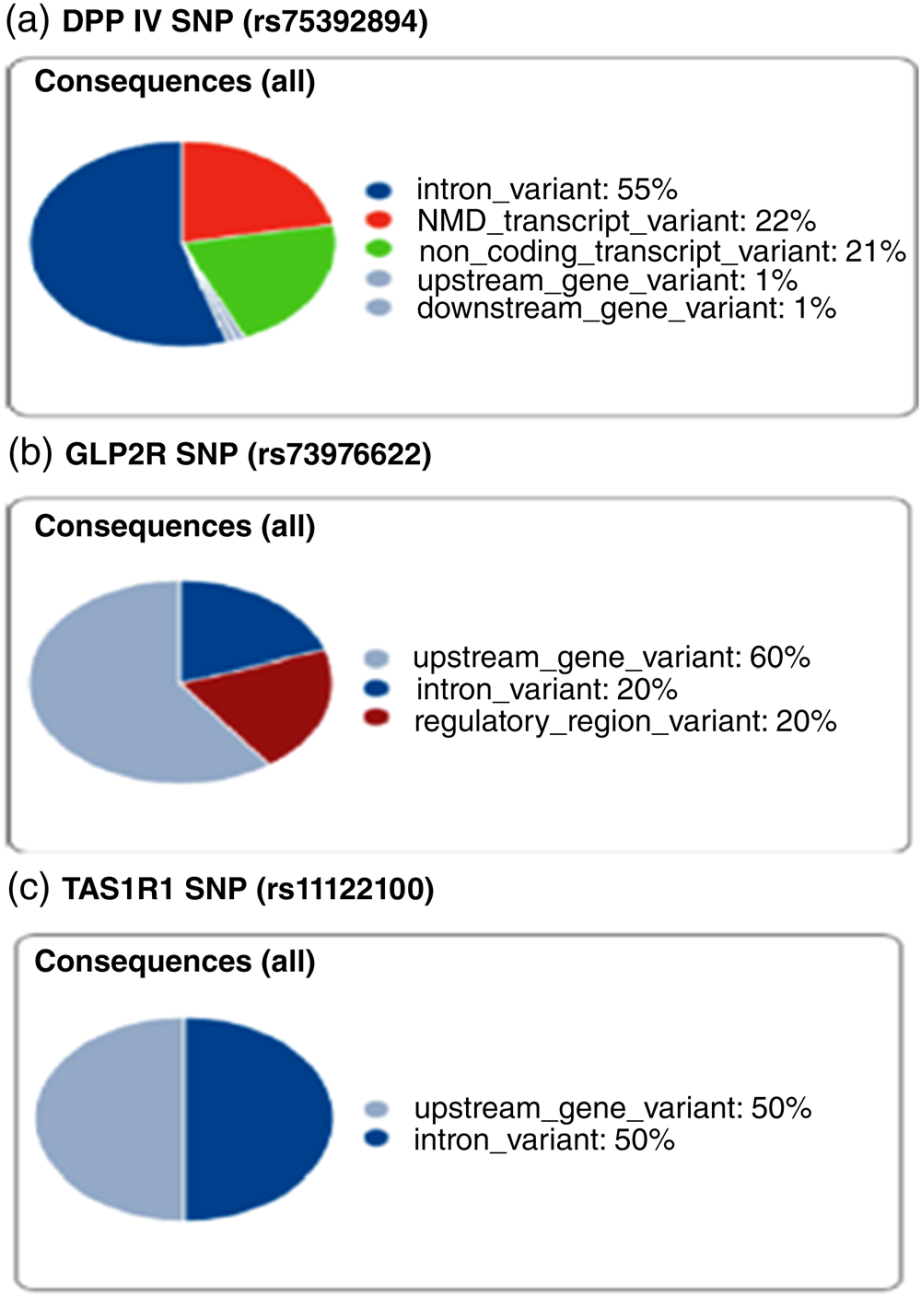
GWAS analysis of DNA samples from both stunted and non‐stunted children revealed SNPs in genes that could be directly linked to GLP‐2. Variant consequences of SNPs in the DPP IV gene (a) were largely in the intron region (55%), while those in the GLP2R (b) and TAS1R1 gene (c) were largely in the upstream region (60% and 50%, respectively).

We additionally evaluated the genetic contribution to variability of circulating levels of GLP‐2 using an African population‐specific array to perform an exploratory GWAS in both stunted and non‐stunted children. Our analysis revealed over 16,000 polymorphisms that were associated with circulating GLP‐2 levels at a threshold of 1 × 10^−2^. No SNPs were associated with the proglucagon gene, the gene encoding GLP‐2, but modest associations were seen between circulating GLP‐2 concentrations and *DPP IV* (*P* = 8.98 × 10^−3^) as well as *GLP2R* (*P* = 5.04 × 10^−3^) polymorphisms, potentially altering GLP‐2 degradation and downstream signalling. A modest association between GLP‐2 and the taste receptor (*TAS1R1*) was also observed (*P* = 3.36 × 10^−3^), reflective of the role taste receptors have in the release of glucagon‐like peptides. Using a more stringent *P*‐value of *P* ≤ 9.90 × 10^5^, a total of 55 genes with 94 SNPs were obtained. Although none of these genes could be directly linked to GLP‐2 release, some could be linked to cell proliferation (*JCAD*, *RIOX2*, *EPS8*) and metabolism (*DERA*, *INO80*, *ECE1*, *ATP6V0E1*). Ninety percent of the SNP variants observed, including in the *DPP IV*, *GLP2R* and *TAS1R1* genes fell in the non‐coding regions (Figure 9) with a few genes exhibiting consequences on the 3′‐untranslated and synonymous region.

## 4 DISCUSSION

The enteroendocrine system secretes peptide hormones in response to meal‐related stimuli. Nutrient stimulated GLP‐2 release can be direct, through enteral stimulation of the L‐cells, or indirect, through mechanisms requiring engagement of neural and endocrine circuits (Drucker & Yusta, 2014). Our findings showed that GLP‐2 levels decreased progressively in stunted children designated non‐responders even though immunofluorescence staining showed the presence of EEC cells. Genetic analysis did not show any definitive variation that could account for the variability seen in GLP‐2, though there was preliminary evidence of genetic contributions to DPP IV and thus GLP‐2 half‐life.

There is widespread concern about the specificity of GLP‐2 ELISAs, as reliable measurement of GLP‐2 levels in plasma is by radioimmunoassay (Hartmann et al., 2000). The ELISA used to detect GLP‐2 levels in this study showed high levels of circulating GLP‐2 and this led us to question whether there was any cross‐reactivity with other proglucagon‐derived products. We tested five synthetic peptides using the ELISA kit and found much lower detection of GLP‐1, oxyntomodulin and GRPP. However, we were unable to exclude the contribution of major proglucagon fragment (MPGF) to circulating GLP‐2 levels. Using other samples collected in children from the same community as our study participants, we have been able to establish that the ELISA results from the Millipore kit are highly correlated with radioimmunoassay measurements done at the University of Copenhagen in Denmark, validating our findings on the progression of circulating GLP‐2 in non‐responsive stunted children.

Among the secretory cell types, Paneth and goblet cell depletion has been shown to be a prominent feature of environmental enteropathy, but less is known about changes in enteroendocrine and tuft cells in enteropathy and malnutrition (Hodges et al., 2021). Goblet, Paneth and endocrine cell loss has also been shown in an adult patient with autoimmune enteropathy (Al Khalidi et al., 2006) as well as in paediatric patients with autoimmune polyendocrine–candidiasis–ectodermal dystrophy (APECED). In contrast, enteroendocrine cells are present in paediatric patients with inflammatory bowel disease, celiac disease, lymphocytic colitis and autoimmune disorders without endocrinopathy (Posovszky et al., 2012). Immunofluorescence staining of tissue obtained from malnourished children showed that all but one sample stained clearly for chromogranin A, a general marker of enteroendocrine cells. The antibodies used for FFAR3, TAS1R1 and GPR119 receptor immunofluorescence have been used in a number of publications (Abdelli et al., 2019; Little et al., 2014; Markovics et al., 2020; Xie et al., 2022; Zhou et al., 2016). However, since the antibodies for a large number of commercially available GPCRs have proven to be non‐specific (Beermann et al., 2012; Bouressam et al., 2018; Hafko et al., 2013; Michel et al., 2009; Yu & Hill, 2013), and we were unable to provide specificity data, the immunofluorescence data for FFAR3, TAS1R1 and GPR119 receptors should be interpreted with caution. Although the specificity of the antibodies used requires confirmation, the co‐existence of this staining with immune‐reactive chromogranin is consistent with its localisation on enteroendocrine cells and provides supporting evidence that the blunted levels of GLP2 in stunted children are not due primarily to a loss in enteroendocrine cells.

GLP‐2 is a potent intestinal trophic peptide with various effects that include an increase in the length of the crypt–villus axis of the intestinal mucosa, suppression of apoptosis of intestinal cells, an increase of blood flow in the mesenteric vessels, decrease of gastrointestinal motility and improvement of the adaptive response to intestinal mucosal damage caused by inflammation (Orhan et al., 2018). Although transcriptomic and peptidomic profiling of human enteroendocrine cells has been done (Roberts et al., 2019), to our knowledge, we have performed the first correlation between circulating GLP‐2 concentrations and the human gut transcriptome. Transcriptomic analysis of tissue obtained from non‐responsive stunted children showed numerous gene transcripts were positively correlated with GLP‐2 levels and were largely centred around metabolism, vesicular transport and intestinal barrier function and not around secretion of GLP‐2, as release is controlled by changes to intracellular calcium levels (Kuhre et al., 2017). GLP‐2 has been shown to affect glucose metabolism as the activation of its receptors, GLP2R, in hypothalamic proopiomelanocortin neurons is required for the enhancement of insulin‐mediated suppression of hepatic glucose production and gluconeogenesis (Baldassano et al., 2016). Vesicular transport is of importance as the multivesicular pathway is the primary means of degrading transmembrane proteins in a cell, linking its function to processes such as nutrient uptake and signalling downregulation (Jones et al., 2012). Vesicular transporters also ensure the efficient delivery of lipids to target organelles and consequently play a role in cell homeostasis (Thelen & Zoncu, 2017). The expressed genes which highly correlated with circulating GLP‐2 were *SLC25A37* (mitoferrin) and *LRG1*. Mitoferrin is a mitochondrial ion transporter that functions as an essential iron importer for the synthesis of mitochondrial haem and iron–sulphur clusters and is involved in erythropoiesis (Bell et al., 2021), while leucine‐rich α‐2 glycoprotein 1 (LRG1) is a glycoprotein that is constitutively expressed by hepatocytes and neutrophils. LRG1 has been reported to be involved in multiple human conditions including diabetes and inflammatory disorders (Camilli et al., 2022).

GLP‐2 has also been shown to promote intestinal barrier function through multiple effects that decrease the permeability of the epithelial barrier (Brubaker, 2018).

Functional analysis further emphasised the role that these gene transcripts play in relation to nutrient sensing and in particular the downstream effects of GLP‐2. Gene transcripts necessary for the differentiation and maturation of secretory cells were identified in the set of genes correlated with GLP‐2 (*ATOH1*, *CDX1*, *CDX2*, *ELF3*, *FZD5*, *HES1*), but no transcripts required for enteroendocrine cell differentiation (*MATH1*, *NGN3*, *BETA2*, *Pax4*) or those directly related to GLP‐2 synthesis were present. Of particular interest were transcription factors ATOH1 and HES1, as active Notch signalling stimulates expression of HES1, a potent repressor for transcription factors ATOH1 and NEUROG3. ATOH1 is essential for the differentiation of secretory cells into specific enteroendocrine cell types while HES1 negatively regulates goblet cell differentiation (Beumer et al., 2020). Goblet cell populations usually follow distinct trajectories as they differentiate and mature, evidenced by the presence of typical markers such as ATOH1, FCGBP and CLCA1. However, some intercrypt goblet cells have been shown to express HES1 (Nyström et al., 2021) with gene ontology enrichment analysis showing that HES1 positive goblet cells demonstrated enrichment of genes associated with lipid and amino acid metabolism, detoxification and intestinal absorption. Recent developments have also suggested that HES1 also plays a role in the regulation of inflammatory responses (Guo et al., 2018). EECs are part of a population of rarer cell types that play crucial roles in tissue maintenance but cannot always be detected by bulk RNA sequencing as they only make up 1% of all epithelial cells. Single cell transcriptomics may be a better way of detecting the presence of such rarefied gene transcripts (Kummerlowe et al., 2022).

GWAS is commonly used to identify differences in polymorphism allele frequencies between study subjects and has gained importance as it can aid in identifying major drivers in disease association studies. GWAS analysis was done on both stunted and non‐stunted children and the findings correlated with baseline GLP‐2 levels. GWAS are usually carried out on a large scale but as this study was exploratory in nature, the sample size was significantly less than what is typically needed in GWAS to achieve statistical power and did not allow for definitive case–control comparison. However, there were some interesting genomic findings when correlated with GLP‐2. SNPs can contribute to changes in the genomic sequence, either in the coding (exon), or intergenic or non‐coding (intron) regions (Vallejos‐Vidal et al., 2020). Consistent with findings in most GWAS analysis, 90% of the SNP variants correlated with GLP‐2 fell in the non‐coding regions, which do not affect the structure of gene products. However, these variants can act by regulating expression levels of genes through their accumulation in DNA regulatory elements or disruption of transcription factor binding sites (Cano‐Gamez & Trynka, 2020). We had initially hypothesised that SNPs in the proglucagon gene might be responsible for the reduced GLP‐2 levels seen in malnourished children, but the GWAS data did not show any association of sequence variation in the proglucagon gene. However, modest associations were seen between circulating GLP‐2 levels and genes for proteins known to interact with GLP‐2 such as TAS1R1, GLP2R and DPP IV. Glucagon‐like peptide‐2‐producing L‐cells have been shown to express *umami* receptors, which are composed of TAS1R1–TAS1R3 heterodimers (Said & Kaunitz, 2016), and this could account for the correlation between circulating GLP‐2 and TAS1R1. The GLP2R SNP could possibly affect the downstream effects of GLP‐2, but it is unlikely that it would affect circulating GLP‐2 concentrations. DPP IV has been shown to proteolytically inactivate GLP‐2, resulting in GLP‐2 (3–33) and a major proglucagon fragment (Janssen et al., 2013). The DPP IV SNP observed in our population (rs75392894) has not been previously reported in PubMed at the time of writing this paper and we believe this is the first report on this DPP IV variant in children with malnutrition. A study by Bohm et al. showed that a DPP IV gene variant (rs6741949) interacted with body adiposity and negatively affected glucose‐stimulated GLP‐1 levels, insulin secretion and glucose tolerance (Bohm et al., 2017). Though these two DPP IV SNPs are different , both gene variants had similar consequences and it is plausible that the SNP identified in our cohort could have an effect on GLP‐2. However, we have no further evidence to support this finding as the two SNPs were found to not be linked using linkage disequilibrium testing. It must be noted, though, that the SNP reported by Bohm et al. could not be found in African populations and this could possibly explain the absence of linkage. A review of literature did not show any genome wide analysis associated with plasma GLP‐2 concentrations, but a reference to genetic determinants of circulating glucose‐dependent insulinotropic peptide (GIP) and GLP‐1 was found (Almgren et al., 2017). That study was able to identify six genome‐wide significant loci associated with circulating insulin, glucagon and GIP levels in or near the solute carrier family 5 member 1 (*SLC5A1*), gastric inhibitory peptide receptor (*GIPR*), ABO blood group (*ABO*), GLP‐2 receptor (*GLP2R*), coagulation factor XIII A (*F13A1*) and homeobox D1 (*HOXD1*) genes. Taking into consideration that GLP‐1 and GLP‐2 are both derived from the same proglucagon gene and are released in equimolar amounts from the gastrointestinal tract (Wismann et al., 2018), we checked for similarities in SNPs between those seen by Almgren et al. and our findings. No identical SNPs were found in our cohort, but SNPs in the *GLP2R* and *F13A1* genes were seen.

We have been able to show that EECs are present in gut tissue of stunted children, with GLP‐2 levels lower in stunted children at the time non‐response is determined. It must be noted that GLP‐2 levels in our Zambian population are still much lower than in other populations, even in the absence of disease (Mutanen & Pakarinen, 2017), and there is need to further explore the meal‐stimulated gut hormonal responses in malnourished populations so as to develop specific interventions.

## AUTHOR CONTRIBUTIONS

Study concept and design—Paul Kelly, Beatrice Amadi, Madusha Peiris, Geoffrey Kwenda, Ellen Besa. Acquisition, analysis and interpretation of data—Mizinga Jacqueline Tembo, Chola Mulenga, Monica Mweetwa, Naheed Choudhry, Kanta Chandwe, Chad Storer, Richard Head, Beatrice Amadi, Talin Haritunians, Dermot McGovern, Madusha Peiris, Ellen Besa. Statistical analysis—Ellen Besa, Monica Mweetwa, Chad Storer, Richard Head, Talin Haritunians, Dermot McGovern, Paul Kelly. Obtained funding—Paul Kelly, Beatrice Amadi. Study supervision—Paul Kelly, Beatrice Amadi, Geoffrey Kwenda. Initial drafting of the manuscript—Ellen Besa and Paul Kelly. Critical revision of the manuscript was done by all the authors. All authors had access to the study data and approved and reviewed the final manuscript. All authors agree to be accountable for all aspects of the work in ensuring that questions related to the accuracy or integrity of any part of the work are appropriately investigated and resolved, and all persons designated as authors qualify for authorship and all those who qualify are listed.

## CONFLICT OF INTEREST

The authors declare no competing financial interests.

## Supporting information

Statistical Summary Document

## Data Availability

The datasets generated and analysed during the study are available from the corresponding author on reasonable request. Fastq data relating to stunted children have been deposited in GEO under accession number 162630.
